# Yield formation at different nodes in the ratoon season of “forage–grain ratoon rice” under cutting time and stubble height

**DOI:** 10.3389/fpls.2025.1630992

**Published:** 2025-08-26

**Authors:** Panpan Gai, Yuanwei Chen, Xin Sun, Hongjing Chen, Desheng Yang, Miaofei Ren, Lei Liu, Weiqin Wang, Huabin Zheng, Qiyuan Tang

**Affiliations:** ^1^ School of Agronomy, Hunan Agricultural University, Changsha, China; ^2^ Crop Research Institute, Hunan Academy of Agricultural Sciences, Changsha, China

**Keywords:** grain yield, ratoon rice, stubble height, cut time, node position, regenerated bud

## Abstract

**Introduction:**

Based on “forage–grain ratoon rice,” the planting pattern of a high-grain-yield ratoon crop (RC), supplemented by fodder, can be regulated by the cutting time and stubble height.

**Methods:**

In 2021 and 2022, using Xiangliangyou 900 as the experimental material, field trials were conducted to investigate differences in the yield formation of regenerated tillers from different nodal positions under varying cutting times (i.e., 10 and 30 days after heading, T10 and T30, respectively) and stubble heights (i.e., 10 and 30 cm, H10 and H30, respectively).

**Results:**

The results showed that the average grain yield of T10 was 80.48% higher than that of T30, while the average grain yield of H30 was 21.77% higher than that of H10. Analysis revealed that the higher yield of T10 could be attributed to the panicle per square meter and the grain filling, while the higher yield of H30 could be attributed to the panicle per square meter. Analysis also revealed that the panicle per square meter and the grain filling of regenerated tillers at different nodes in T10 were higher than those in T30, while the yield performance of regenerated tillers at different nodes in H was more complex. Further analysis revealed that, under the H30 treatment, the grain yield generally followed the order D4 > D3 > D2, with D3 and D4 contributing approximately 84.80% of the total yield on average. In contrast, under the H10 treatment, D3 exhibited a higher yield than D4, with D3 contributing approximately 58.05% of the total yield. Notably, the D3 yield under H10 was higher than that under H30, while D4 showed the opposite trend.

**Discussion:**

Reasonable cutting times and stubble heights are important factors to increase the yield of the ratoon season.

## Graphical Abstract

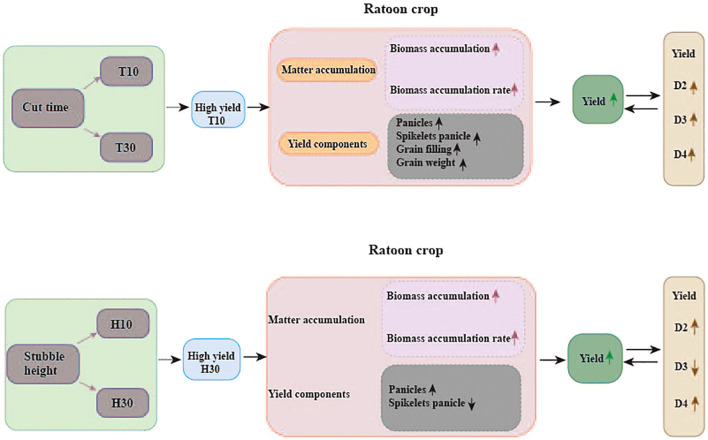

## 1 Introduction

China has a large population, and the development of herbivorous animal husbandry is rapid. However, land resources are limited, and there is a high demand for roughage. There is a lack of large-scale grassland and grass planting foundations, and the acquisition of grass mainly relies on imports (Dong et al., 2020). By 2030, the demand for livestock products is expected to reach 294 ton, increasing the pressure on feed grain consumption. In 2022, the feed grain consumption accounted for 48% of China’s total grain consumption, surpassing edible grain by nearly 15% (Qi et al., 2024). Therefore, the coordinated development of grain, economic crops, and feed systems has become a research focus. At present, due to the low efficiency of grain cultivation and the large outflow of the rural population, there is a shortage of labor force. Rice planting has changed from the previous “double season” to a “single season” and even abandoned land, resulting in a downward trend in the rice planting area (Chen et al., 2022). Ratoon rice, as one of the rice planting modes, is more labor-saving and cost-effective compared with double cropping rice. However, the quality of the first-season rice is poor and the yield of the ratoon season is low, limiting the promotion and application of the ratoon rice planting mode for human food. Through early harvesting of ratoon rice in the first season, the growth period of the ratoon season can be extended, which can solve the issue of low yield in this season and provide stable and quality-guaranteed green roughage for herbivores (Zou et al., 2020).

Ratoon rice, known as twice harvesting after one sowing, has drawn much attention and has been widely practiced in China (Wang et al., 2021). Ratoon crop (RC) can save land preparation costs, seeds, and crop establishment, thereby substantially increasing farmer profits (Yang et al., 2022). The period of main crop (MC) harvesting is an important factor that affects the RC and influences the sheath emergence of regenerating buds and the reproductive process (Duan et al., 2020). Non-structural carbohydrates (NSC) are generally considered as the major subclass of the organic reserves mobilized during regrowth (White, 1973). Ratoon rice is related to the NSC in rice stubbles over and above the role of the root system. During the growth and development of the first-season rice crop, various starches and sugars accumulate in the leaves and stalks of rice and are subsequently transferred into the developing grain after flowering (Turner and Fund, 1993). The more carbohydrates remaining after the first season’s grain harvest, the higher the grain yield of ratoon rice (Xu et al., 2002). However, there needs to be more information on the differences between different cutting times and stubble heights on the yield attributes at different nodes.

The stubble height for MC harvesting is an essential factor that affects the RC yield and determines whether the axillary buds at different nodes on the rice stubble are retained or not (Xiong, 2018). A decrease in stubble height tended to reduce the total number of spikes, but increased the number of spikes from the basal nodes, the spike size, and the growth duration (Xu et al., 2021). Harrell et al. (2009) demonstrated that, as the stubble height was reduced from 40 to 20 cm, the growth point of the axillary buds was reduced and the seed yield of ratoon rice increased, mainly due to the increase in the number of grains in the spike. It has also been shown that higher stubble retains more ratoon buds from the upper nodes, which increases the RC yield and the yield stability (Cao et al., 2020). Jiang et al. (2005) reported that the two-line hybrid combination Pei’ai 64S/E32 increased the yield of ratoon rice by 53.52% when compared to the low stubble height (10 cm) and high stubble height (30 cm). The critical average daily temperature for normal flowering of indica rice is 22°C, and the critical average daily temperature for stable and reliable fertility of ratoon rice above 70% is 21°C (Huang et al., 1989). Overseas studies have shown that lowering the stubble height extended the maturity time of ratoon grains by 1–10 days and delayed the maturity time of grains (Tarpley et al., 2008). Domestic studies have shown that the fertility period of ratoon grains was extended by 3–5 days for every 5 cm reduction in the height of the cut stakes within the range 5–35 cm (Zou et al., 2023). However, determining a reasonable stubble height is related to the light and temperature resources, as well as to a variety of regeneration characteristics. Tang and Qing (2023) reported that the ratoon season in Hunan should be fledged safely by September 15 in order to avoid the effects of chilling winds. Therefore, low retention stakes may cause the ratoon season to encounter the hazards of low temperatures. In terms of regional distribution, the machine-harvested high retention stakes (30–35 cm) adopted at this stage are suitable for the middle and lower reaches of the Yangtze River (Hunan), rice areas with relatively scarce light and temperature resources during the ratoon season. The short fertility period of high retention stakes can ensure the safe flush of spikes (Lin et al., 2024).

Based on the demand for forage and the increase in the yield of ratoon rice, we propose a new planting mode for ratoon rice, i.e., the dual-purpose ratoon rice (forage–grain ratoon rice, FG-RR) planting mode, to balance the feed demand and the high yield of ratoon rice. The appropriate combination of cutting time and stubble height becomes the critical technical link of this cultivation model. Although the feasibility of the forage and food model has been reported, there are fewer reports on the effects of different cutting times and stubble heights on the differences in yield formation at various nodes of ratoon rice. The purpose of this study was to clarify the yield and yield-related traits of ratoon tillers at different nodes under different stubble heights and cutting times in the FG-RR mode, assuming that the yield of ratoon tillers at different nodes responds differently to stubble height and cutting time.

## 2 Materials and methods

### 2.1 Site description

A field experiment was conducted from 2021 to 2022 at Jinjing Town (113°38′ E, 28°53′ N), Changsha County, Hunan Province. The site is located within the main subtropical monsoon climate zone. Ratoon rice is the leading rice cropping system in the area and is usually planted from March to October. The fields are left fallow for the remaining non-rice season. Before transplanting, five soil cores were collected obliquely from the 0- to 20-cm soil layer and were analyzed for the main soil properties. In 2021–2022, the soil was clay loam, with pH values of 5.78 and 5.53, organic matter of 18.7 and 19.9 g kg^−1^, total N of 1.5 and 1.8 g kg^−1^, fast-acting phosphorus of 18.3 and 18.9 mg kg^−1^, and available potassium of 122.3 and 117.7 mg kg^−1^, respectively.

### 2.2 Experimental design and crop management

**Figure 1:**
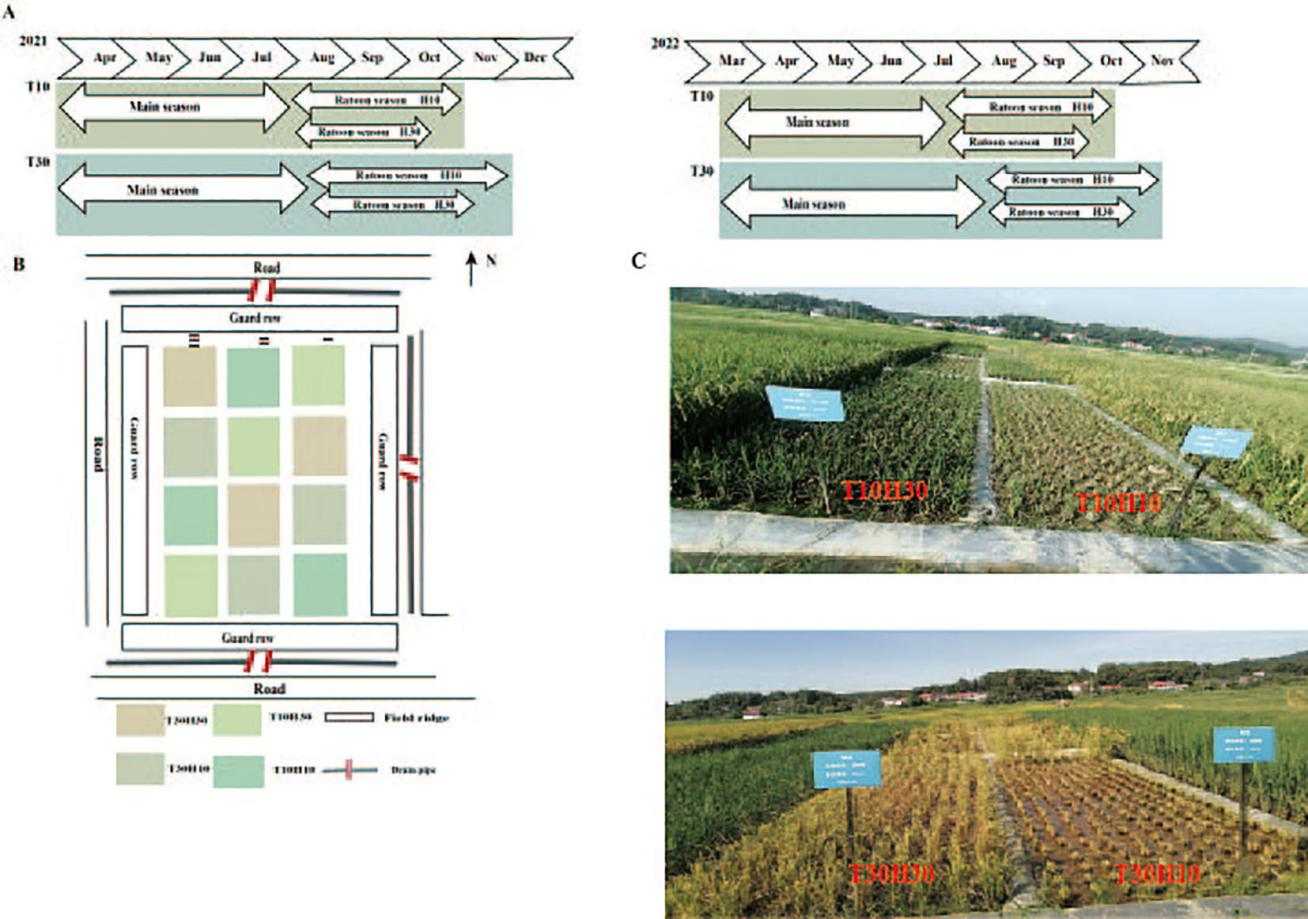
**(A)** Growing season of crops in different treatments. **(B, C)** Detailed field layout maps.

XLY900 was used as the material in this study (hybrid rice bred from Guangxiang 24S × R900). As a “forage–food” dual-purpose ratoon rice type, this variety shows better silage yield and quality in the first season and better yield and rice quality in the ratoon season (preliminary research of the group). The study employed a randomized complete block design with three replications, with each plot measuring 20 m^2^ (4 m × 5 m). Two cutting time treatments were established: MC at 10 days after heading (T10) and MC at 30 days after heading (T30, the traditional harvesting time). Two stubble height treatments were implemented, with free combinations among all treatment pairs: 10-cm stubble height (H10) and 30-cm stubble height (H30). Furrows between the different experimental plots were covered with a plastic film to prevent interference from fertilizers and moisture. All furrows were also covered with a plastic film to minimize leakage between the experimental plots. Rice seeds were sown on April 5, 2021, and on March 23, 2022. Two seedlings per hill (13.3-cm × 30-cm hills) were manually transplanted from May 5, 2021, to April 22, 2022. For MC, 195 kg N ha^−1^ was split-applied with 50% as basal (1 day before transplanting), 30% at early tillering (7 days after transplanting), and 20% at panicle initiation. For RC, 75 kg N ha^−1^ was applied as bud-promoting fertilizer (N_B_) at 15 days after the heading of MC and 75 kg N ha^−1^ applied as seedling fertilizer (N_T_) at 1 day after the harvest of MC. Phosphorus at 40 kg ha^−1^, as calcium superphosphate, was applied at basal. Potassium of 120 kg ha^−1^, as potassium chloride, was split equally and applied at basal and at 15 days after the heading of MC. For MC, a water depth of 3–5 cm was maintained from transplanting to maturity, except for the drainage at the maximum tillering stage to reduce unproductive tillers and 7 days before the first-season harvest (or longer, if the weather is unfavorable). For RC, a water depth of 2–3 cm was maintained until maturity, except for the drainage at the ratoon season harvest 7 days before (or longer, if the weather is unfavorable). Weeds, diseases, and insects were controlled to avoid yield loss. The growth season, the field layout design, and the field display diagram of the crops (two seasons) under different cutting periods and stubble heights in the main season are shown in **Figure 1**.

### 2.3 Sampling and measurements

#### 2.3.1 Meteorological data

A small meteorological station was used to collect and record the temperature (maximum, minimum, and average), solar radiation, and rainfall in the test area. The meteorological station (Em50, METER Group, Inc., Pullman, WA, USA) is located within 100 m of the test field.

#### 2.3.2 Growth stage

The key growth stages of the main season and the ratoon season, including the sowing date, the transplanting date, the panicle initiation (PI), the heading date (HD), and the maturity date (MD) of the main season were recorded, as well as the HD and MD of the ratoon season. PI was determined by the shoot apex pubescence, HD at 80% panicle exertion, and MD at 90% (MC) or 95% (RC) grain yellowing.

#### 2.3.3 Agronomic traits and growth characteristics

At both the heading and maturity stages of the RC, 12 representative hills (0.479 m^2^) were randomly selected from each plot for measurement. Subsequently, the roots were cut off, the ratoon shoots peeled off from where they were attached to the main-season rice stalks, the main-season rice stalks separated from the ratoon shoots, and the ratoon shoots divided into leaves, stem sheaths, and panicles. The dry weight of each organ was determined after oven drying at 80°C to constant weight. Biomass accumulation (total dry weight, TDW) was calculated as the sum of the dry weight of each organ.

#### 2.3.4 Grain yield and yield components

At the maturity stage of the RC, a 4-m^2^ area with uniform growth was selected from the center of each plot as the yield measurement zone. The grains were threshed and dried, and empty grains were removed using a fan. The rice grains were then weighed after reaching moisture equilibrium, and the moisture content was measured using an automatic digital moisture meter (DMC-700, Seedburo, Chicago, IL, USA) to determine the moisture content of the rice grains (average of three measurements). The total yield was calculated using the standard calculation method, with the rice yield calculated based on 14% moisture absorption.

In addition, another 12 hill (0.479 m^2^) samples were taken during the maturity stage of the ratoon season (excluding the three border hills). RC regenerating tillers at maturity were grouped by nodes [i.e., nodes 2, 3, and 4 (D2, D3, and D4, respectively) from the top at a cutting height of 30 cm; D3 and D4 at a cutting height of 10 cm] and then divided into spikelets. The panicles were hand-threshed and separated from the solid and empty grains through aqueous sorting. A subsample of 30 g solid grains and 3 g empty grains from three subsamples was used to count the number of spikelets. The dry weights of rachis and filled and empty spikelets were determined after drying at 80°C to constant weight.

The following formulas were used for calculation: Number of spikelets per panicle = spikelets m^−2^/panicles m^−2^; harvest index (HI) = filled spikelet weight/total dry weight × 100%; and crop growth rate (CGR) = biomass accumulation/growth duration (Yang et al., 2022).

### 2.4 Data analysis

Statistical analysis of the data was performed with SPSS software 21.0 (SPSS Inc., Chicago, IL, USA). All data were statistically analyzed using two-way ANOVA. Statistical significance between the treatments was examined with the Duncan method at the *p* < 0.05 (LSD_0.05_) probability level. Graphical representation of the data was performed using GraphPad Prism version 9.0 software.

## 3 Results

### 3.1 Weather conditions

**Figure 2:**
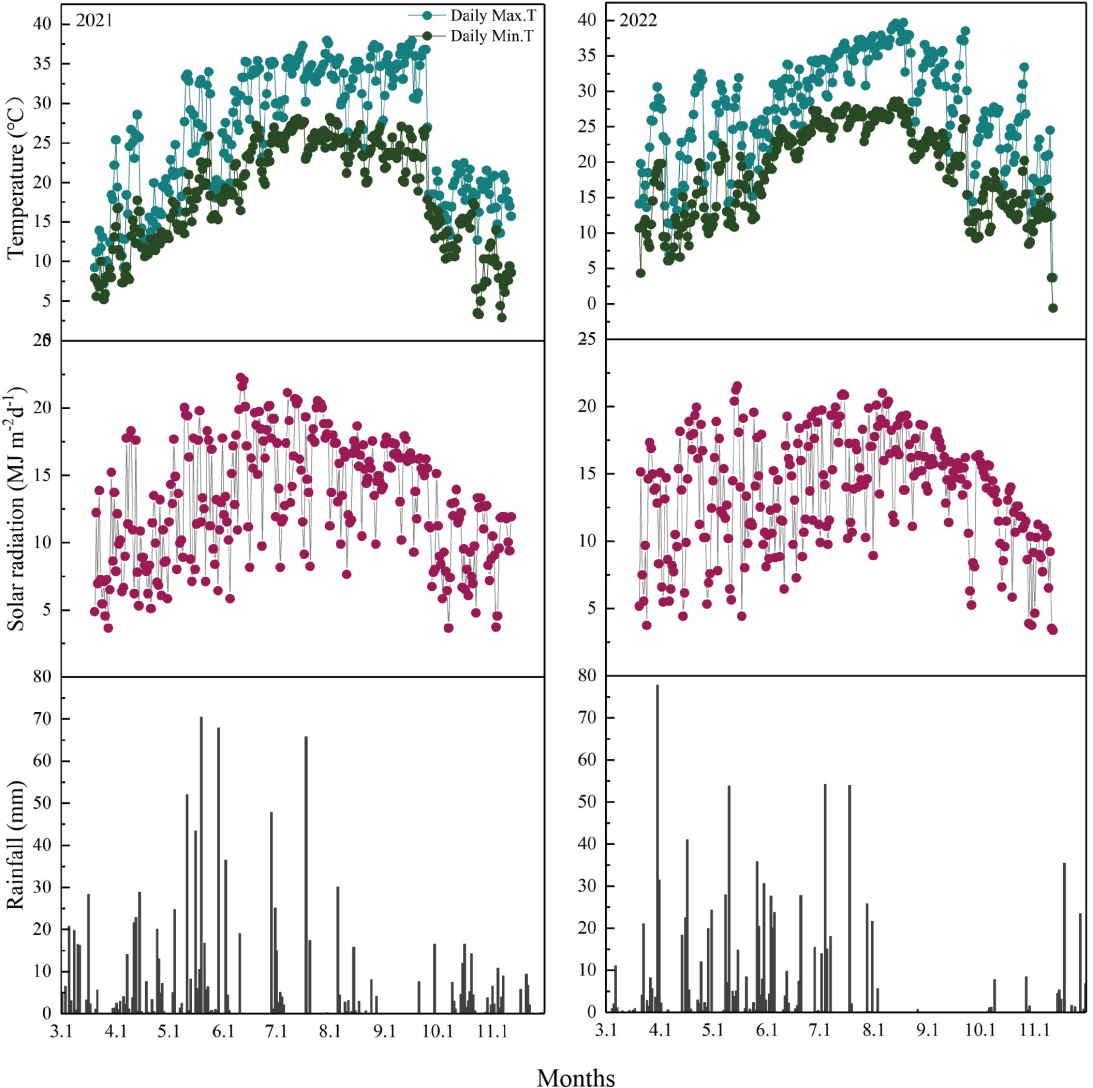
Daily maximum and minimum temperatures, solar radiation, and precipitation during the rice growing season from the transplanting of the main crop to the maturity of the ratoon crop at Changsha County, Hunan Province, Central China, in 2021 (*left*) and 2022 (*right*) (Gai et al., 2024).

**Figure 2** shows the daily maximum and minimum temperatures, solar radiation, and precipitation during the rice growing season from the transplanting of MC to the maturity of RC. During the main and ratoon seasons, the average daily maximum temperatures were 27.10°C and 28.39°C; the minimum temperatures were 19.24°C and 19.71°C; the annual average solar radiation values were 13.48 and 14.15 MJ m^−2^; and the total precipitation values were 961.60 and 901.80 mm, respectively. The average daily temperatures in RC were 2.80°C higher in 2022 than in 2021. The total solar radiation in RC also varied between the two years, which was 39.36 MJ m^−2^ higher in 2022 than in 2021. In contrast, the rainfall was lower in 2022 than 2021.

### 3.2 Growth duration

In 2021, the MC cutting times for T10 and T30 were on August 2 and August 22, respectively. In 2022, the MC cutting times for T10 and T30 were on July 24 and August 13, respectively. For the ratoon season, the harvesting times in 2021 and 2022 were as follows: T10H10, November 12 and October 20; T10H30, October 25 and October 10; T30H10, December 4 and November 14; and T30H30, November 23 and November 3 (**Figure 1A**) (Gai et al., 2024).

### 3.3 Yield and yield components of the different nodes

There are differences in the biomass of the main-season straw at different cutting stages under the same cutting height (**Supplementary Figure S1**), and the biomass of the main-season straw in T30 was significantly higher than that in T10. When the stubble heights were 10 and 30 cm, the average biomass values of rice straw were 39,874.1 and 37,317.2 kg hm^−2^ in 2021 and were 26,367.3 and 23,651.1 kg hm^−2^ in 2022, respectively, with significant differences between them.

**Figure 3:**
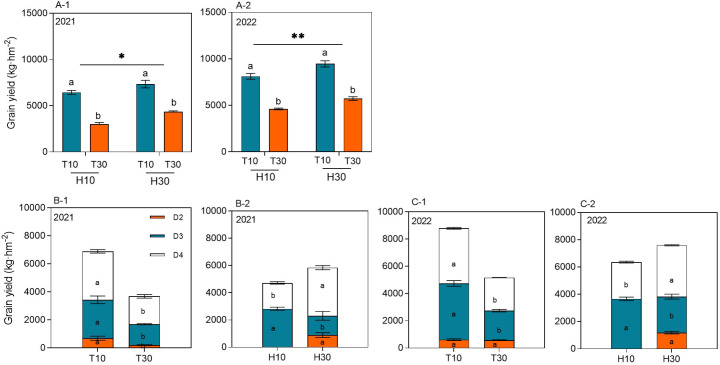
**(A-1–C-2)** Grain yield of ratoon tillers in the second, third, and fourth nodes (*D2*, *D3*, and *D4*, respectively) from the top of the ratoon crop under different cut time and stubble height interactions and single-factor conditions in 2021 and 2022. *Different lowercase letters* denote statistical differences in the cut time and stubble height treatments according to the least significant difference (LSD) test (0.05). Data are the mean ± standard error. *T10*, cut 10 days after heading in the first season; *H10*, stubble height of 10 cm; *T30*, cut 30 days after heading in the first season; *H30*, stubble height of 30 cm. * indicates p<0.05 (5% significance level), ** indicates p<0.01 (1% significance level).

The MC seed yield ranged from 3,004.98 to 9,466.37 kg hm^−2^ at different cutting times, stubble heights, and years. The yield of RC under the intercropping conditions at different cutting times and stubble heights reached significant levels in both years. Under the same conditions of H10 or H30, the regrowth season yield of T10 was significantly higher by 114.44% or 76.07% and by 68.74% or 65.23% in 2021 and 2022, respectively, compared with T30. When T10 and T30 were considered, the ratoon season yield of H30 increased by 23.76% and 19.37% in 2021 and 2022, respectively, compared with H10 (**Figures 3**A-1, A-2). The cutting time and the stubble height significantly affected the yield of ratoon tillers at different nodes in 2021 and 2022 (**Figures 3**B-1, B-2, C-1, C-2). The yield of ratoon tiller plants of D3 and D4 significantly increased under T10 treatment compared with T30, with increases in yield of 83.75% and 89.83% and of 74.25% and 67.41%, respectively, in both years. However, T30, in addition to improving the yield of D2 ratoon tiller plants, also showed differences between years. The yield of D3 ratoon tiller plants was significantly increased in H10 treatment compared with H30, with 98.87% and 38.73% increases in yield in both years. However, the yield of D4 ratoon tiller plants was significantly decreased in the H10 treatment compared with H30, with 46.18% and 28.28% reductions in yield in both years. However, there were no D2 ratoon tiller plants in H10 treatment as only H30 had D2 preserved.

### 3.4 Panicles and panicles at different nodes

**Figure 4:**
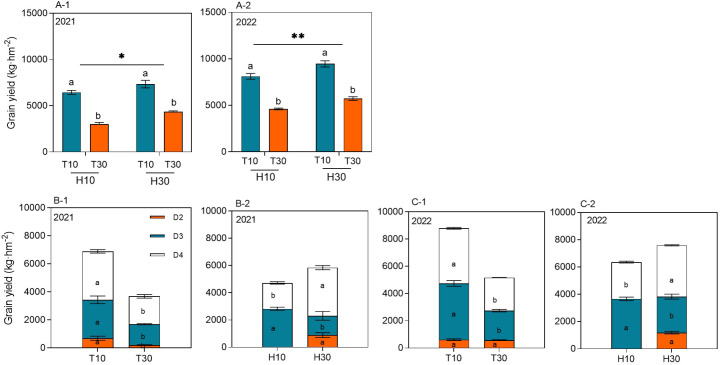
**(A-1–C-2)** Panicles per square meter in the second, third, and fourth nodes (*D2*, *D3*, and *D4*, respectively) from the top of the ratoon crop under different cut time and stubble height interactions and single-factor conditions in 2021 and 2022. *Different lowercase letters* denote statistical differences in the cut time and stubble height treatments according to the least significant difference (LSD) test (0.05). Data are the mean ± standard error. *T10*, cut 10 days after heading in the first season; *H10*, stubble height of 10 cm; *T30*, cut 30 days after heading in the first season; *H30*, stubble height of 30 cm. ** indicates p<0.01 (1% significance level).

There were significant differences in the number of ratoon spikes at the different nodes under different cutting time and stubble height interactions (**Figure 4**). Under the same conditions of H10 or H30, the number of ratoon spikes was significantly higher in T10 than in T30 in 2021 and 2022. The number of ratoon spikes was considerably higher in H30 than in H10 in 2021 and 2022, also considering T10 and T30 as a whole (**Figures 4A-1, A-2**). There were differences in the effect of cutting time or stubble height on the number of ratoon spikes at different nodes in 2021 and 2022 (**Figures 4B-1, B-2, C-1, C-2**). The number of ratoon spikes of D4 was significantly increased under T10 treatment compared with T30 by 26.50% and 12.97% in the two years. In contrast, the number of ratoon spikes of D3 was significantly higher, with the absence of any significant difference. However, as can be seen from **Figures 4B-1, C-1**, the number of D2 ratoon spikes under T10 treatment was greater than that under T30 treatment. The number of D3 ratoon spikes was significantly increased under H10 treatment compared with H30 by 53.94% and 39.63% in both years. However, the number of D4 ratoon spikes was significantly decreased under H10 treatment compared with H30 by 48.27% and 44.40% in both years. However, the ratoon buds of D2 under the H10 treatment were mowed as only D2 was preserved in H30.

### 3.5 Ratooning ability and ratooning ability at different nodes

**Figure 5:**
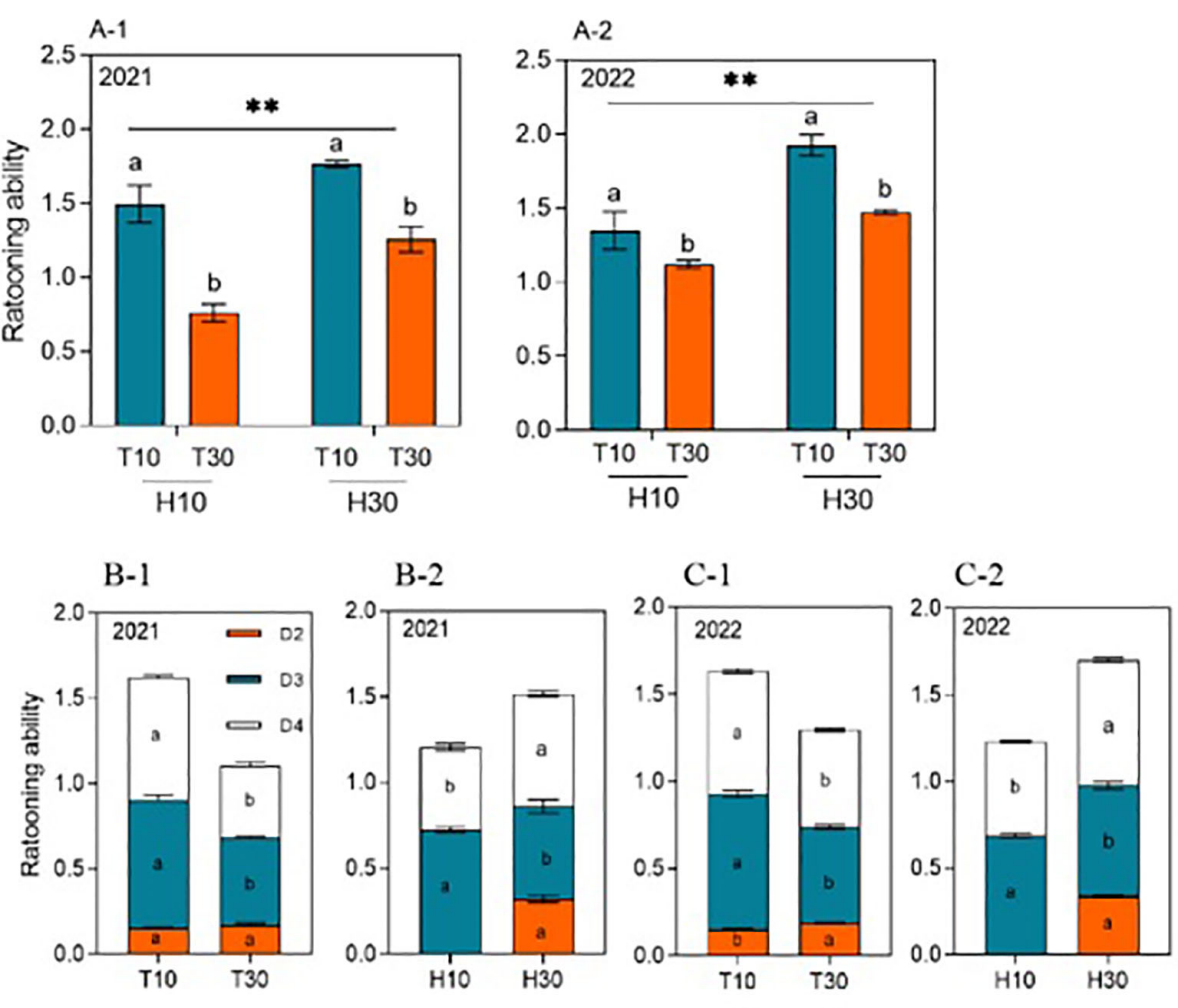
**(A-1–C-2)** Ratooning ability in the second, third, and fourth nodes (*D2*, *D3*, and *D4*, respectively) from the top of the ratoon crop under different cut time and stubble height interactions and single-factor conditions in 2021 and 2022. *Different lowercase letters* denote statistical differences in the cut time and stubble height treatments according to the least significant difference (LSD) test (0.05). Data are the mean ± standard error. *T10*, cut 10 days after heading in the first season; *H10*, stubble height of 10 cm; *T30*, cut 30 days after heading in the first season; *H30*, stubble height of 30 cm. ** indicates p<0.01 (1% significance level).

Interestingly, the cutting time and stubble height reciprocal yields and single-factor patterns of change in the ratoon tiller yield and regeneration force at different nodes were similar (**Figure 5**). Under the same conditions of H10 or H30, the regeneration force of T10 was significantly higher than that of T30 in 2021 and 2022. Also considering T10 and T30 as a whole, the regeneration force of H30 was significantly higher than that of H10 in 2021 and 2022 (**Figures 5A-1, A-2**). Differences were observed in the effect of cutting time or stubble height on regeneration at the different nodes in 2021 and 2022 (**Figures 5B-1, B-2, C-1, C-2**), with D3 and D4 regeneration being significantly enhanced under T10 treatment compared with T30, increasing by 47.18% and 40.79% and by 69.18% and 26.91% in 2021 and 2022, respectively. However, T30 enhanced the D2 regeneration, which also showed differences between years. D3 regeneration was significantly increased under H10 treatment by 34.31% and 7.47% in 2021 and 2022 compared with H30. However, D4 regeneration was significantly decreased under H10 treatment by 26.91% and 25.55% in both years compared with H30. Similarly, D2 regeneration was present only under H30.

### 3.6 Yield components of RC

The yield components of RC are shown in **Tables 1** and **2**. There were differences in the yield components of the different nodes in the ratoon season under the interactions of cutting times and stubble heights in 2021 and 2022. In 2021, the number of grains at different nodes was more affected by the interaction, whereas in 2022, this was also more affected by the 1,000-grain weight at different nodes. Overall, in 2021 and 2022, T10H10 and T30H10 had less effect on the yield components of D3 and D4, while T10H30 and T30H30 were more affected by year. In 2021, there were no significant differences between T10H30 and T30H30 on the percentage of nodal nodules and 1,000-kernel weight at different nodes, but the number of spikes of D4 was significantly higher than those of D3 and D2. In 2022, the number of spikes of the effects of T10H30 and T30H30 on the number of grains in spikes at different nodes was the same as those in 2021, while the 1,000-grain weight of D4 under the T10H30 and T30H30 treatments increased by 0.87% and 3.56% compared with those of D3 and D2, respectively. In 2021 and 2022, the yield component factors of D2 were significantly higher than those of T30 under the T10 treatment, but the yield component factors of D3 and D4 were affected by year. Some differences were observed. In 2021, T10 and T30 had no significant effect on the number of grains in spikes and the grain filling of D3 and D4, but T10 significantly increased the grain filling of D3 and D4 by 64.92% and 66.04%, respectively. In 2021 and 2022, the number of grains in spikes and the grain filling of D2 under the H30 treatment were significantly higher than those of H10, while the number of grains in spikes of D3 and D4 under the H10 treatment was significantly higher than that of H30.

**Table 1:** Yield-related traits of ratoon tillers in the second, third, and fourth nodes (D2, D3, and D4, respectively) from the top of the ratoon crop (RC) at different cut stages and stubble heights in 2021.

Cutting time	Stubble height	Inverse node	Spikelets per panicle	Grain filling (%)	Grain weight (mg)
T10	H10	D3	156a	58.9a	24.0a
		D4	154a	57.5b	24.1a
	H30	D2	106b	59.4a	23.5a
		D3	101bc	61.0a	23.2a
		D4	130a	57.5a	23.1a
T30	H10	D3	168a	36.3a	21.9a
		D4	172a	35.3a	22.2a
	H30	D2	88b	35.9a	24.4a
		D3	99b	36.5a	24.4a
		D4	132a	34.6a	23.6a
Cutting time					
T10		D2	53a	29.68a	12.22a
		D3	129a	59.98a	23.60a
		D4	142a	58.33a	23.56a
T30		D2	44b	17.93b	11.75b
		D3	133a	36.37b	22.96a
		D4	152a	35.13b	23.17a
Stubble height					
H10		D2	0b	0.00b	0.00b
		D3	162a	47.60a	23.20a
		D4	163a	46.40a	23.09b
H30		D2	97a	47.62a	23.96a
		D3	100b	48.75a	23.84a
		D4	130b	47.07a	23.49a

Different lowercase letters represent statistical differences between the different node positions under different cutting times or stubble heights using the least significant difference (LSD) test (0.05).

**Table 2:** Yield-related traits of ratoon tillers in the second, third, and fourth nodes (D2, D3, and D4, respectively) from the top of the ratoon crop (RC) at different cut stages and stubble heights in 2022.

Cutting time	Stubble height	Inverse node	Spikelets per panicle	Grain filling (%)	Grain weight (mg)
T10	H10	D3	164a	67.5a	24.1a
		D4	167a	66.8a	23.1b
	H30	D2	104b	71.1a	22.8b
		D3	102b	70.5a	23.2b
		D4	127a	72.5a	23.6a
T30	H10	D3	141a	48.5a	23.6a
		D4	141a	47.7a	23.1a
	H30	D2	95bc	56.9a	22.5b
		D3	100b	55.6a	23.1a
		D4	112a	50.7b	23.3a
Cutting time					
T10		D2	52a	35.53a	11.38a
		D3	133a	68.98a	23.63a
		D4	142a	90.75a	23.57a
T30		D2	47b	28.47b	11.24b
		D3	120b	52.07b	23.37a
		D4	127b	49.18b	23.28a
Stubble height					
H10		D2	0b	0.00b	0.00b
		D3	152a	57.98b	23.84a
		D4	149a	81.07a	23.13a
H30		D2	99a	64.00a	22.62a
		D3	101b	63.07a	23.14b
		D4	119b	61.68a	23.71a

Different lowercase letters represent statistical differences between the different node positions under different cutting times or stubble heights using the least significant difference (LSD) test (0.05).

### 3.7 Other yield attributes of RC

The CGR, biomass accumulation, HI, and RC regeneration are listed in **Tables 3** and **4**. There were a number of differences between the different cutting stage and stubble height interactions in 2021 and 2022. In 2021, there were significant differences between the different treatments for each of the yield attributes of the post-sprouting CGR, the pre-sprouting MH-HD (which is the accumulation of biomass during the cutting and heading stages), and the ratooning ability (RA), which were highest for T10H30, whereas there were no significant differences between HI. After heading, biomass accumulation was not significantly different between T10H10 and T10H30. The other treatments were significantly lower than both, with T30H10 being the lowest. In 2022, there were no significant differences in the yield attributes between T10H10 and T10H30, as well as between T30H10 and T30H30; however, the yield attributes of T10H10 and T10H30 were significantly higher than those of T30H10 and T30H30 (except for regeneration). In 2021 and 2022, the yield attributes under the different cutting time treatments showed T10 > T30, while the effect of stubble height on the yield attributes was strongly influenced by year. H10 significantly reduced the HI and RA by 14.73% and 5.36% and by 33.63% and 38.21%, respectively. However, in 2022, there was no significant difference between the post-sprouting CGR and biomass before tasseling; only in 2021 were they significantly different, where the yield attributes showed H30 > H10.

**Table 3:** Crop growth rate (CGR), biomass accumulation from the main crop (MC) harvest (MH) to the ratoon crop (RC) heading (HD) and from HD to the RC harvest (RH), harvest index (HI), and ratooning ability (RA) of XLY900 in the 2021 ratoon season.

Cutting time	Stubble height	CGR (g m^−2^ day^−1^)		Biomass (g m^−2^)		HI (%)	RA
		MH-HD	HD-RH	MH-HD	HD-RH		
T10	H10	15.03ab	14.20b	884.14b	752.62a	38.67a	1.50b
	H30	17.07a	17.13a	1,012.03a	736.16a	40.37a	1.77a
T30	H10	11.90c	3.00d	700.25d	170.12c	24.33c	0.76d
	H30	13.83bc	4.13c	803.70c	235.89b	31.91b	1.26c
Cutting time							
T10		16.06a	15.66a	1,503.95a	744.39a	39.52a	1.63a
T30		12.84b	3.56b	948.09b	203.00b	28.12b	1.01b
Stubble height							
H10		13.45a	8.59b	792.20b	461.37a	31.50b	1.13b
H30		15.45a	10.63a	907.86a	486.02a	36.14a	1.51a

Different lowercase letters represent statistical differences between the different node positions under different cutting times or stubble heights using the least significant difference (LSD) test (0.05).

**Table 4:** Crop growth rate (CGR), biomass accumulation from the main crop (MC) harvest (MH) to the ratoon crop (RC) heading (HD) and from HD to the RC harvest (RH), harvest index (HI), and ratooning ability (RA) of XLY900 in the 2022 ratoon season.

Cutting time	Stubble height	CGR (g m^−–2^ day^−1^)		Biomass (g m^−2^)		HI (%)	RA
		MH-HD	HD-RH	MH-HD	HD-RH		
T10	H10	18.90a	19.53a	1,022.77a	840.57a	42.53b	1.35b
	H30	21.43a	21.80a	1,122.96a	915.17a	45.84a	1.92a
T30	H10	9.70b	9.50b	612.20b	446.02b	39.62c	1.12c
	H30	11.17b	8.07b	729.66b	379.64b	40.72bc	1.47b
Cutting time							
T10		20.18a	20.67a	1,072.87a	877.87a	44.18a	1.64a
T30		10.43b	8.78b	670.93b	412.83b	40.17b	1.30b
Stubble height							
H10		14.31a	14.52a	817.49a	643.29a	41.08b	1.23b
H30		16.30a	14.93a	926.31a	647.40a	43.28a	1.70a

Different lowercase letters represent statistical differences between the different node positions under different cutting times or stubble heights using the least significant difference (LSD) test (0.05).

### 3.8 Role of the yield traits in yield

**Figure 6:**
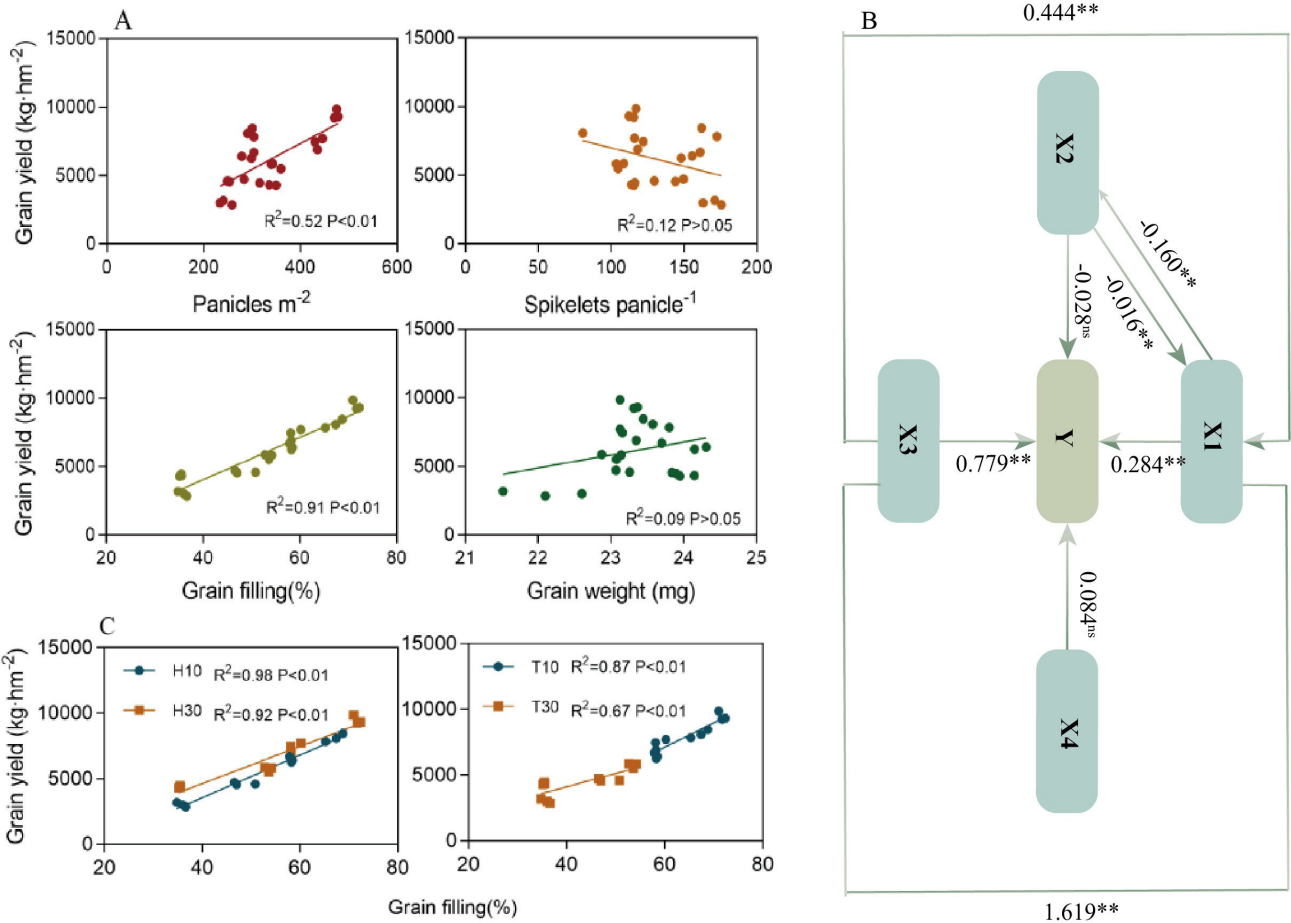
Linear correlation **(A, C)** and pathway analysis **(B)** to determine the major factors affecting yield. *Y*, yield; *X1*, panicles per square meter; *X2*, spikelets per panicle; *X3*, grain filling (in percent); *X4*, grain weight (in milligrams). The *black line* and the corresponding data indicate the direct contribution to yield based on the pathway analysis, where *two asterisks* and *one asterisk* indicate significant differences at the 0.01 and 0.05 levels, respectively. *ns* indicates no significant difference. ** indicates p<0.01 (1% significance level).

Linear correlation analysis (**Figure 6A**) showed that the grain yield was positively and significantly correlated (*p* < 0.01) with the number of spikes and the fruiting percentage, but not correlated (*p* > 0.05) with the number of grains in a spike and the 1,000-grain weight. Further analysis of the relationship between yield and fruiting percentage under the cutting time (T) and stubble height (H) conditions revealed that both were significantly and positively correlated, but the slope of T10 was greater than those of the other three (**Figure 6C**). Analyses of the yield components revealed that, particularly under the same H condition, T10 had a significantly higher grain filling at different nodes than T30 (**Tables 1**, **2**). The linear correlation, excluding the effect of spike number and 1,000-grain weight on yield, made it difficult to determine whether spike number or grain filling played a greater role in yield. In the pathway analysis (**Figure 6B**), the coefficient of the direct effect of X1 on yield was 0.284**, while that of X3 was 0.799**. It was concluded that the contribution of X3 to yield was greater than that of X1. However, it is interesting to note that the effect of X1→X3→Y (1.619**) was much greater than that of X3→X1→Y (0.444**), suggesting that the change in X3 is still based on X1, which in turn suggests that the effect of X1 on yield is relatively large.

### 3.9 RDA and PLS-PM analysis

**Figure 7:**
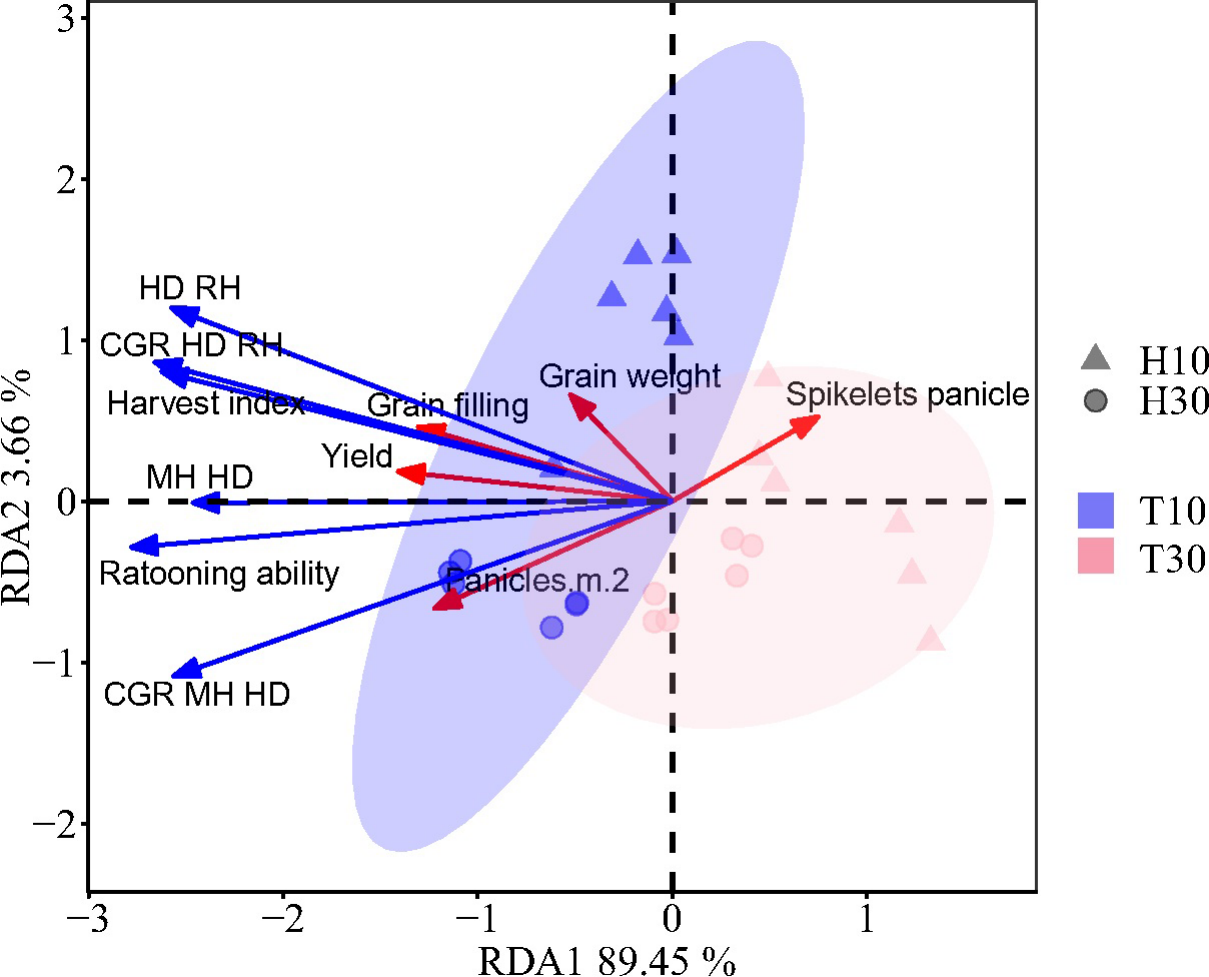
Redundancy analysis of yield and the yield components in the ratoon season and growth-related traits of regenerated crops. *Red arrows* denote the grain yield and yield components. *Blue arrows* indicate the growth-related traits of the ratoon crop. *MH-HD*, accumulation of biomass during the cutting and heading stages; *HD-RH*, accumulation of biomass during the mature period from the heading stage to the regeneration season; *CGR (MH-HD)*, rate of biomass accumulation during the cutting and heading stages; *CGR (HD-RH)*, rate of biomass accumulation during the mature period from the heading stage to the ratoon season.

**Figure 8:**
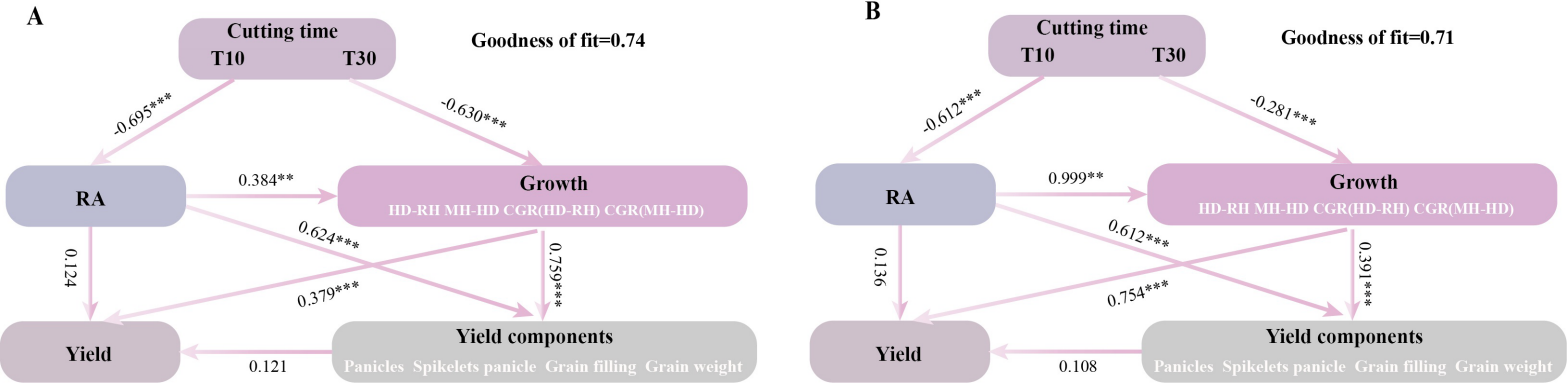
**(A, B)** Partial least squares path modeling (PLS-PM) analysis describing the relationship between growth correlation, regeneration ability, yield, and the composition factors of regenerated crop yield at different cutting times, stubble heights, and ratoon crops. *RA*, regenerative ability; *MH-HD*, accumulation of biomass during the cutting and heading stages; *HD-RH*, accumulation of biomass during the mature period from the heading stage to the regeneration season; *CGR (MH-HD)*, rate of biomass accumulation during the cutting and heading stages; *CGR (HD-RH)*, rate of biomass accumulation during the mature period from the heading stage to the ratoon season. *One asterisk* and *two asterisks* denote significance at the probability levels 0.05 and 0.01, respectively. *** indicates p < 0.001 (0.1% significance level).

Redundancy analysis (RDA) indicated a positive correlation between the growth-related traits and the grain yield, except for the number of grains per spike (**Figure 7**). Partial least squares path modeling (PLS-PM) analysis showed that both T and H had a significant negative regulatory effect on the growth-related traits, with meridian coefficients of −0.630 and −0.281, respectively. However, T had a highly significant negative regulatory effect on regenerative power, while H had a highly significant positive regulatory effect (**Figure 8**). Although there was no significant direct effect between RA and yield, RA directly affected the growth-related traits, thereby increasing yield. Both T and H directly regulated yield positively. It was found that the correlation between RA and growth had a highly significant positive regulatory effect on the yield components, while the yield components had no significant direct effect on yield. This indicates that the treatment was not entirely positively regulating the yield components, as confirmed by the RDA.

## 4 Discussion

The yield and yield attributes showed inconsistencies between the 2 years of the ratoon season. Overall, in 2022, strong RA improved the biomass accumulation and the rate of accumulation and thus the panicle number and grain filling, resulting in a higher yield in the ratoon season than that in 2021. The main reason may be that the effective cumulative temperature and solar radiation were higher in 2022 than in 2021 (**Figure 1**). Previous research by this group showed that, when harvesting whole plant silage as the primary purpose, it is preferable to adopt the cutting treatment of 25–30 days after spike flushes and 10–20 cm stubble heights in the first season, at which time the silage quality is better; when harvesting rice grains in the ratoon season as the primary purpose, it is preferable to adopt the cutting treatment of 10–15 days after spike flushes and 20–30 cm stubble heights in the first season, which results in a higher yield in the ratoon season (Chen et al., 2022). In the present study, under the same H treatment, the ratoon season yields of T10 were significantly higher by 114.44% and 76.07% in 2021 and by 68.74% and 65.23% in 2022 compared with T30. When T10 and T30 were considered as a whole, the ratoon season yields of H30 in 2021 and 2022 increased by 23.76% and 19.37%, respectively, compared with H10 (**Figures 3**A-1, A-2).

The effects of the T and H single factors on the yields of different nodes were analyzed. T10 was found to significantly increase the yields of D2, D3, and D4 compared with T30, which may be due to the stronger root vigor of rice 7 days after flushes, which is more favorable to the regeneration of dormant buds at the lower nodes. Moreover, the earlier the first-season rice is mowed, the more abundant are the temperature and light resources during the regeneration period, which is more favorable to the regeneration of grouting and filling and the formation of a high yield (Peng et al., 2020). However, the effect of H on the yields of the different nodes was relatively complicated, with the yields of D2 and D4 under H30 treatment being significantly higher than that under H10; the effect on the yield of D3 showed the opposite trend. However, the increase of the D2+D4 yield was much lower than the decrease of the D3 yield, which finally led to the yield of H30 being higher than that of H10 (Yi et al., 2005a). The high retention of stubble was conducive to the restoration and maintenance of the vigor of the main-season root system, and the main-season roots are still the main part of the ratoon rice root system, thus providing a good opportunity for the D4 yield to be improved and maintained. The first-season roots are still the main body of the ratoon rice root system, thus providing nutrients for the D4 axillary buds preferentially (Yi et al., 2005b). Higher stubble retains the axillary buds at the upper nodes, and the residual stubble N and carbohydrate concentrations in the D2 node are higher than those in the D3 and D4 nodes, so that nutrients from the stalk itself are preferentially supplied to the axillary buds in the D2 node. However, the total yield was not high due to the early differentiation of young spikes, the short fertility period, and the low number of glumes per spike, which made it difficult to form large spikes (Jiang et al., 2002). The effect of D2 apical dominance inhibited the growth of axillary buds at D3 nodes (Domagalska and Leyser, 2011; Yu et al., 2022). The fact that the D4 node yield was lower under H10 than H30 suggests that apical dominance inhibition has the principle of proximity inhibition. Further analyses to determine the reason for H30 reducing the yield of the D3 node are needed. Interestingly, the D3 yield was significantly higher under H10 than that in H30 (Yang et al., 2022). It might be that there was no competitive inhibition of high buds and that low buds gave full play to their potential (Huang et al., 2009). The growth point of axillary buds was reduced, the fertility period of ratoon rice was prolonged, the maturity period was prolonged, and the seed yield of ratoon rice was increased (an increase in the number of grains per spike). It was also found that both the D3 and D4 yield components were higher than that of D2 in the same rice plant under the H30 treatment (Dong et al., 2013). The plant’s own NSC content, in particular starch, is mainly stored in the basal stem internodes, and the NSC in the top third stem internode was significantly higher than that in the top first and second stem internodes (Xiong et al., 2000). The results of the above study were analyzed, and it was found that approximately 50% of the nutrients absorbed by the old roots is supplied to the low buds, while only around 30% is supplied to the high buds. Approximately 74% of the nutrients absorbed by the ratoon new roots is supplied to the low buds, while around 18% is supplied to the high buds, which may be the reason for the yield components of D3 and D4 being greater than those of D2 under the H30 treatment.

It is generally accepted that the grain yield in RC is largely affected by the panicle number, which is highly dependent on the survival of the ratoon buds (Tang et al., 2002). Studies have shown that the number and the rate of regeneration spikes have a highly significant positive correlation with the ratoon season yield (He et al., 2019). A higher stubble dry weight left behind after harvesting of the MC provides more carbohydrates, which favors the growth of ratoon buds (Xu et al., 2015), increasing the number of spikes per unit area in the ratoon season. However, a large number of ratoon buds died after the heading of the MC, mainly due to the increase of the light energy allocated to seeds and the decrease of the light energy allocated to the ratoon buds during the irrigation period of the MC, which led to the death of the ratoon buds after the heading period (Xu et al., 2000). In addition, cutting at the flush stage allows more photosynthetic products to be retained in the stem sheath, thus providing sufficient nutrient supply for regeneration bud germination and growth (Torres et al., 2020). Under the same conditions of H, the regeneration ability and the number of panicles in T10 were significantly higher than those in T30, which is consistent with Xu et al. (2021). Considering T10 and T30 as a whole, the H30 regeneration and number of spikes were significantly higher than those in H10 (**Figures 3**A-1, A-2, **4**A-1, A-2). H30 preserved the axillary buds of the D2 node; therefore, it had absolute difference in the number of spikes and regeneration compared with H10 (**Figures 4**, **5**), while H30 significantly increased the number of spikes and regeneration of D4 and indeed reduced D3. We believe that, with high stubble, the sprouting of high buds is the result of a combination of factors, while the sprouting of low buds is obviously inhibited by high buds; however, changes in any of the environmental conditions or the mother stem may cause a series of chain reactions, which could change the dominant shoot position. In particular, the increase in D4 regeneration is directly related to cytokinins, which are synthesized in the root moving apically to the buds or locally and have a stimulatory effect on lateral shoot growth, in addition to possibly being due to the regeneration of the root system (Thomas, 2002), which promotes the growth of axillary buds.

Stubble height is a crucial agronomic factor that greatly determines the grain yield of RC. At a stubble height of 10 cm, the effects of T10 and T30 on the yield components at different nodes of the same plant were small. However, at a stubble height of 30 cm, the number of spikes, the grain filling, and the 1,000-grain weight were higher in D4 than in D3 and D2 in the T10 and T30 coleoptiles, with the number of spikes being more significant. A second accumulation of NSC in the stalks might have occurred near the maturity of the first-season crop, which is conducive to the growth and development of regenerative root systems (Xu et al., 2021), thus favoring nutrient uptake at the D4 node. The effects of T and H on the grain number, grain filling, and 1,000-grain weight were analyzed, and it was found that T10 favored the increase of D2, D3, and D4 grain fillings, which ranged from 24.80% to 65.25%, from 32.48% to 64.92%, and from 66.04 to 84.53%, respectively, and also significantly increased the number of grains per spike, which may be attributed to the fact that a better combination of temperature and light resources during the differentiation period of young spikes in the ratoon season promotes glume differentiation, which in turn increases the number of grains per spike, resulting in full grain filling due to the abundant resources (Peng et al., 2020). While H30 retained the D2 axillary buds, it also significantly reduced the number of grains per spike in D3 and D4 by 50.66%–62.00% and 24.89%–25.38%, respectively, compared with H10 and had a smaller effect on the D3 and D4 fruiting percentage and 1,000-grain weight, which was mainly due to the fact that lowering the height of the retained stubbles could reduce the growth point of axillary buds, increase the spikelets per panicle, and prolong the growth duration of RC, prolonging its fertility time (Harrell et al., 2009), thereby increasing the source and reservoir capacity and favoring the formation of large spikes. Unlike the first-season rice, the number of spikes per unit area was the dominant factor affecting yield in the ratoon season, with the number of glumes per spike having the second highest effect, while the grain filling and grain weight were relatively stable (Lin et al., 2019). This may partly be explained by the fact that the number of grains per spike in D3 and D4 under the H30 treatment was lower than that in H10, but with the yield still being higher than that in H10.

Furthermore, our study showed that different cutting times and stubble heights would result in different relationships between the RC yield and yield attributes (**Figure 6**). The seed yield was significantly and positively correlated with the number of spikes per square meter and the grain filling, but was not correlated with the number of grains in a spike and the 1,000-grain weight (**Figure 6A**). The pathway analysis further demonstrated that the number of spikes and grain filling had greater direct effects on the yield, with grain filling being the main factor (**Figure 6B**). Further analysis of the single-factor conditions of H and T demonstrated that the slope of T10 was higher than those of others, indicating that the increase in fruiting percentage was mainly related to the earlier cutting (**Figure 6C**). It was also found that H was positively correlated with yield (**Figure 6C**), which is in agreement with the results shown in **Tables 1** and **2**. This is in general agreement with the findings of Xu et al. (2015), showing that a high stubble improved the yield through the combined effects of increasing the effective spike number and the grain filling. The higher grain filling in the high stubble treatment compared with the low stubble was due to the faster germination and the shorter fertility of the upper buds in the high stubble treatment, as well as the higher autumn temperatures at this time of the year, which resulted in a more adequate supply of nutrients to the plant (Xu et al., 2015). However, it should be noted that, while stubble height has a significant impact on the grain filling, the grain filling is primarily influenced by different cutting times. It was found that, in addition to grain filling, the number of spikes had a greater direct effect on yield (**Figures 6A, B**). Pathway analysis showed that there was no significant correlation between the spikelets per panicle and the yield (**Figure 6B**). The PLS-PM analysis revealed that the yield composition factors had no direct effect on yield, indicating that these factors have both positive and negative regulation on the yield (**Figure 8**). RDA found a negative correlation between yield and spikelets per panicle (**Figure 7**). Chen et al. (2023a) reported that the high yield of ratoon rice is mainly dependent on the number of spikes and the grain filling rather than the number of spikelets per panicle (from the results of the group’s 2018 and 2019 trials). The above results are consistent with the results of this experiment. A high yield in ratoon season rice can be achieved by focusing on the number of effective regeneration spikes under the premise of clarifying the appropriate total number of glumes per unit area (Li et al., 2012). Moreover, early cutting and high stubble increase the number of spikes. All these are consistent with the results of our study.

## 5 Conclusions

The accumulation of biomass and the accumulation rate of ratoon rice throughout the reproductive period were higher than those of normal cutting. The increased ratoon rice RA and HI led to an increase in yield, and the increase in total yield was due to the increase in yield at each node, with the yield components at each node being higher in early cutting than in normal cutting. The accumulation of biomass and the rate of accumulation of ratoon rice throughout the reproductive period were higher at high stubble than at low stubble (with the differences being relatively small). While significantly increasing the ratoon rice RA and HI, the increase in yield at D2+D4 nodes was greater than the decrease in yield at the D3 node, ultimately achieving an increase in yield. The number of panicles is the main factor affecting the yield of regenerated rice, and there is a negative correlation between the yield of the regenerated season and the number of grains per panicle. Therefore, coordinating the number of panicles with the number of grains per panicle may be a key factor in achieving yield. The results showed that both early cutting and high stubble are favorable to XLY 900 for achieving high yield.

## Data Availability

The original contributions presented in the study are included in the article/**Supplementary Material**. Further inquiries can be directed to the corresponding authors.

## Supplementary material

Supplementary Figure 1 Straw yield of main-season rice under different cutting times and stubble heights of ‘forage–food’ dual-purpose ratoon rice (Chen et al., 2023b). Different lowercase letters indicate significant difference at P < 0.05 between cutting times the same stubble height. ^*^indicates the total fresh weight of rice straw differences between different stubble height under t-test, ^**^P < 0.01; ^*^P < 0.05; ns: no significant difference.
