# Supplementary figures and images for: Yield formation at different nodes in the ratoon season of “forage–grain ratoon rice” under cutting time and stubble height
Source: Front Plant Sci. 2025 Aug 26;16:1630992. doi: 10.3389/fpls.2025.1630992 (PMC12418450; doi:10.3389/fpls.2025.1630992)

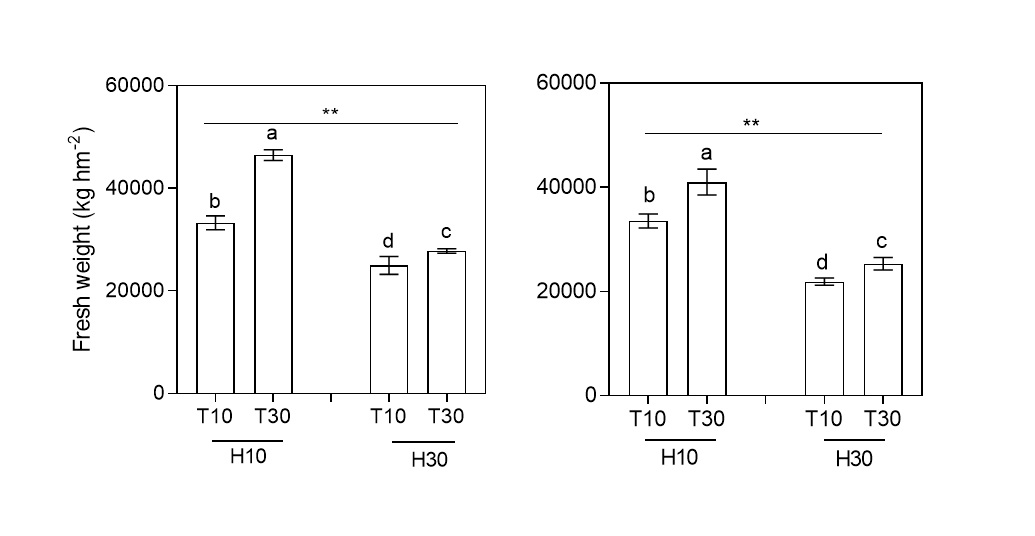

Supplement: Supplementary Figure 1 — Straw yield of main-season rice under different cutting times and stubble heights of ‘forage–food’ dual-purpose ratoon rice (Chen et al., 2023b). Different lowercase letters indicate significant difference at P < 0.05 between cutting times the same stubble height. *indicates the total fresh weight of rice straw differences between different stubble height under t-test, **P < 0.01; *P < 0.05; ns: no significant difference. [file Image1.jpeg]
